# Apparent Non-Canonical Trans-Splicing Is Generated by Reverse Transcriptase *In Vitro*

**DOI:** 10.1371/journal.pone.0012271

**Published:** 2010-08-18

**Authors:** Jonathan Houseley, David Tollervey

**Affiliations:** Wellcome Trust Centre for Cell Biology, University of Edinburgh, Edinburgh, United Kingdom; Victor Chang Cardiac Research Institute, Australia

## Abstract

**Background:**

Trans-splicing, the *in vivo* joining of two independently transcribed RNA molecules, is well characterized in lower eukaryotes, but was long thought absent from metazoans. However, recent bioinformatic analyses of EST sequences suggested widespread trans-splicing in mammals. These apparently spliced transcripts generally lacked canonical splice sites, leading us to question their authenticity. Particularly, the native ability of reverse transcriptase enzymes to template switch during transcription could produce apparently trans-spliced sequences.

**Principal Findings:**

Here we report an *in vitro* system for the analysis of template switching in reverse transcription. Using highly purified RNA substrates, we show the reproducible occurrence of apparent trans-splicing between two RNA molecules. Other reported non-canonical splicing events such as exon shuffling and sense-antisense fusions were also readily detected. The latter caused the production of apparent antisense non-coding RNAs, which are also reported to be abundant in humans.

**Conclusions:**

We propose that most reported examples of non-canonical splicing in metazoans arise through template switching by reverse transcriptase during cDNA preparation. We further show that the products of template switching can vary between reverse transcriptases, providing a simple diagnostic for identifying many of these experimental artifacts.

## Introduction

Reverse transcriptases (RTs) are enzymes that synthesize complementary DNA (cDNA) from an RNA template, and have evolved in retroviruses to convert single stranded viral RNA into double stranded DNA for integration into host genomes. They are an invaluable tool for molecular biology, being used to copy RNA into DNA for analysis by PCR (RT-PCR), microarrays and high throughput sequencing. It is possible to sequence RNA directly, however, most of the experimentally determined RNA sequences, and all high-throughput data, have been generated by RT-based protocols.

RTs lack a proof reading activity (reviewed in [1]) and consequently typically show a fidelity of nucleotide incorporation that is orders of magnitude lower than that of DNA polymerases. This generally causes few problems, as comparison of RNA and genomic DNA sequences allows easy identification of base substitutions. However, less easily detectable sequence errors can be introduced by another intrinsic property of RTs. Retroviral replication is known to require two template switches, where RT ‘jumps’ to another template location without terminating DNA synthesis [2], and this ability is also implicated in high retroviral mutability [3]. Template switching has been repeatedly implicated in the observation of apparent intramolecular splicing events [4], [5], [6], [7], [8], and evidence for its involvement in apparent intermolecular trans-splicing has also been reported [9], although this is disputed [10]. A key observation regarding these apparent splicing events is that they occur between non-canonical splice sites that often share short homologous sequences. This presumably reflects a requirement for homology between the nascent transcript and the acceptor site to allow RT to prime continued cDNA synthesis after template switching [4].

Trans-splicing of common mRNA leader sequences has long been known to occur in trypanosomes, nematode worms and sea squirts (reviewed in [11]), but appeared to be very rare in mammalian cells (reviewed in [12]). Unexpectedly, however, bio-informatic analyses of mammalian transcripts reported large numbers of ostensible trans-splicing events [13], [14], [15], [16]. The observation that trans-spliced products could be detected from almost 50% of human genes [14] provided the key evidence underlying the recent suggestion that trans-splicing is a frequently used method of increasing transcriptome complexity in higher eukaryotes [17]. If real, these trans-splicing events must utilize an as yet undiscovered splicing mechanism as the exons involved mostly lacked canonical splice sites. Notably, however, they often showed short homologous sequences at the donor and acceptor sites [14].

Here we report the development of an *in vitro* system to study the occurrence of template switching events during reverse transcription. Our data greatly extend the range of substrates that can be considered likely to be formed by reverse transcriptase artifact when encountered as part of high throughput sequencing data sets.

## Results

**Figure 1:**
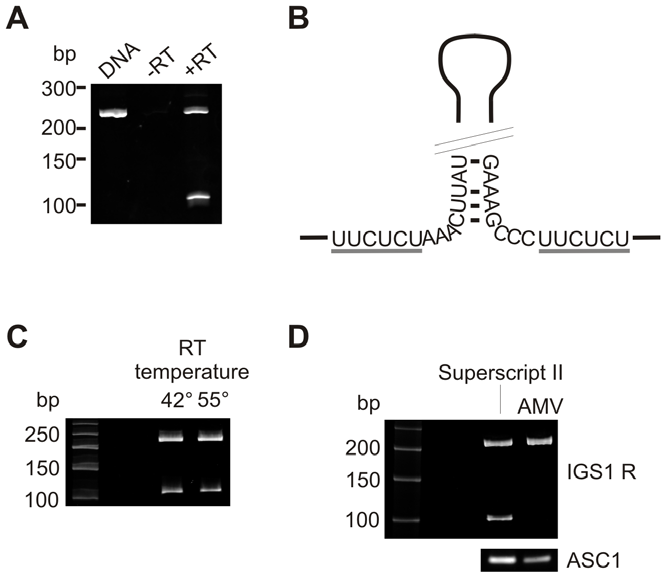
An apparent non-canonical intron in the IGS1 R non-coding RNA. A: 35 cycle RT-PCR across the apparent intron on cDNA synthesized with Superscript II and genomic DNA. cDNA was produced from a *trf4*Δ strain where this non-coding RNA is stabilized. B: Hairpin structure of IGS1 R, short homologous repeats are underlined in grey. C: 35 cycle RT-PCR across the apparent intron on *trf4*Δ cDNA synthesized using Superscript II at 42°C or Superscript III at 55°C. D: 35 cycle RT-PCR across the apparent intron on *trf4*Δ cDNA synthesized using Superscipt II or AMV. Control shows 30 cycle RT-PCR reaction across the *ASC1* mRNA intron on the same cDNA samples.

During the RT-PCR analysis of a yeast non-coding RNA, IGS1 R [18], we detected an apparent splicing event removing a 117 nt intron from about 30% of transcripts (Fig. 1A). Surprisingly, the putative intron lacked conserved sequences normally present at the intron branch point, 5′ and 3′ splice sites, which are highly conserved between yeast pre-mRNAs. It was, however, flanked by two short homologous sequences predicted to lie at the base of a hairpin in the unspliced RNA (Fig. 1B). Previous analyses had suggested that apparent splicing might arise from template switching by RT and we therefore tested whether changing the reverse transcription conditions would alter the result. Increasing the reaction temperature has been reported to suppress template switching [4], [7] but had no effect on the apparent abundance of spliced IGS1 R (Fig. 1C). However, the putative spliced product was observed following RT-PCR using Superscript II (a Moloney Murine Leukemia Virus derived RT) but not with AMV (an Avian Myeloblastosis Virus derived RT) (Fig. 1D). This demonstrated that the apparent splicing of IGS1 R arises from an RT artifact, which is dependent on the specific RT used.

**Figure 2:**
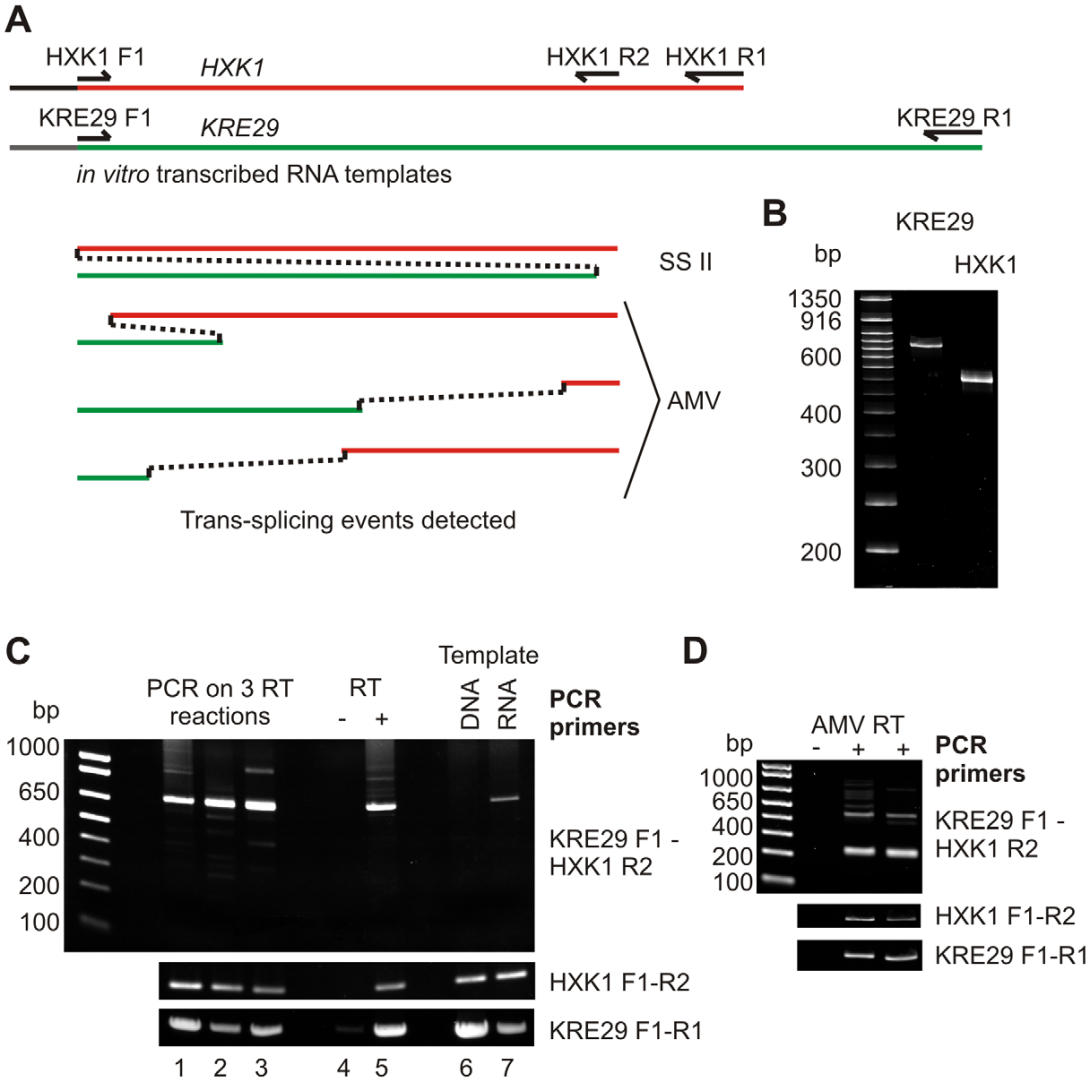
An *in vitro* system for the analysis of apparent trans-splicing. A: *HXK1* and *KRE29* substrate RNAs showing primer locations. Template switching events produced by Superscript II and AMV are indicated. B: Purified substrate RNAs. C: RT-PCR using primers complementary to each RNA on three independent RT reactions (lanes 1–3), and a no RT control (Lanes 4–5). The template for the DNA control (lanes 6–7) was HeLa cDNA with restriction fragments encompassing the entire sequence of the substrate RNAs. Upper panel 35 cycles, other panels 25 cycles. D: PCR reactions performed as in C on cDNA produced with AMV reverse transcriptase. Upper panel 35 cycles; lower panels 25 cycles.

The observation that non-canonical splicing events can reproducibly occur between short repeated sequences lead us to question whether many recently reported trans-splicing events are in fact due to template switching artifacts. Proving that any particular splicing event does not occur at low levels is problematic, so we instead attempted to reproduce apparent trans-splicing using RT *in vitro*. From the five budding yeast trans-splicing events reported by Li et al. (2009), we arbitrarily selected GenBank sequence M14410, a fusion between *KRE29* and *HXK1*, as a substrate for *in vitro* analysis (Fig. 2A). Regions spanning a few hundred base pairs either side of the apparent trans-splicing sites in both genes were amplified from genomic DNA and cloned, providing sequence-verified DNA templates. RNAs were transcribed with T7 RNA polymerase and purified by gel extraction (Fig. 2B). The two individual RNA molecules were mixed, diluted 1∶1000 with HeLa total RNA, and then reverse transcribed from random hexamers using Superscript II. The substrate RNAs were diluted in HeLa total RNA to mimic the high complexity of the RNA population in real RT reactions, and to ensure that template switching was not being driven by the presence of only the donor and recipient.

PCR was performed with primers designed to detect trans-splicing events and this produced the same product in three independent RT reactions performed on three different occasions (Fig. 2C lanes 1–3). Sequencing of this product revealed an apparent trans-splicing event from near the end of the *HXK1* RNA to the middle of the *KRE29* RNA. Formation of this product required RT (Fig. 2C lanes 4–5), and was not a PCR artifact, as it was not amplified from *HXK1* and *KRE29* DNA mixed with HeLa cDNA (Fig. 2C lanes 6–7). Using AMV RT, multiple RT-PCR reactions yielded different products to those observed with Superscript II (Fig. 2D), which were shown to represent at least three different apparent trans-splicing events by sequencing (Fig. 2A). We conclude that both Superscript and AMV reproducibly generate apparent trans-spliced products on the *HXK1* and *KRE29* template pair, but with distinct preferred fusion sites.

**Figure 3:**
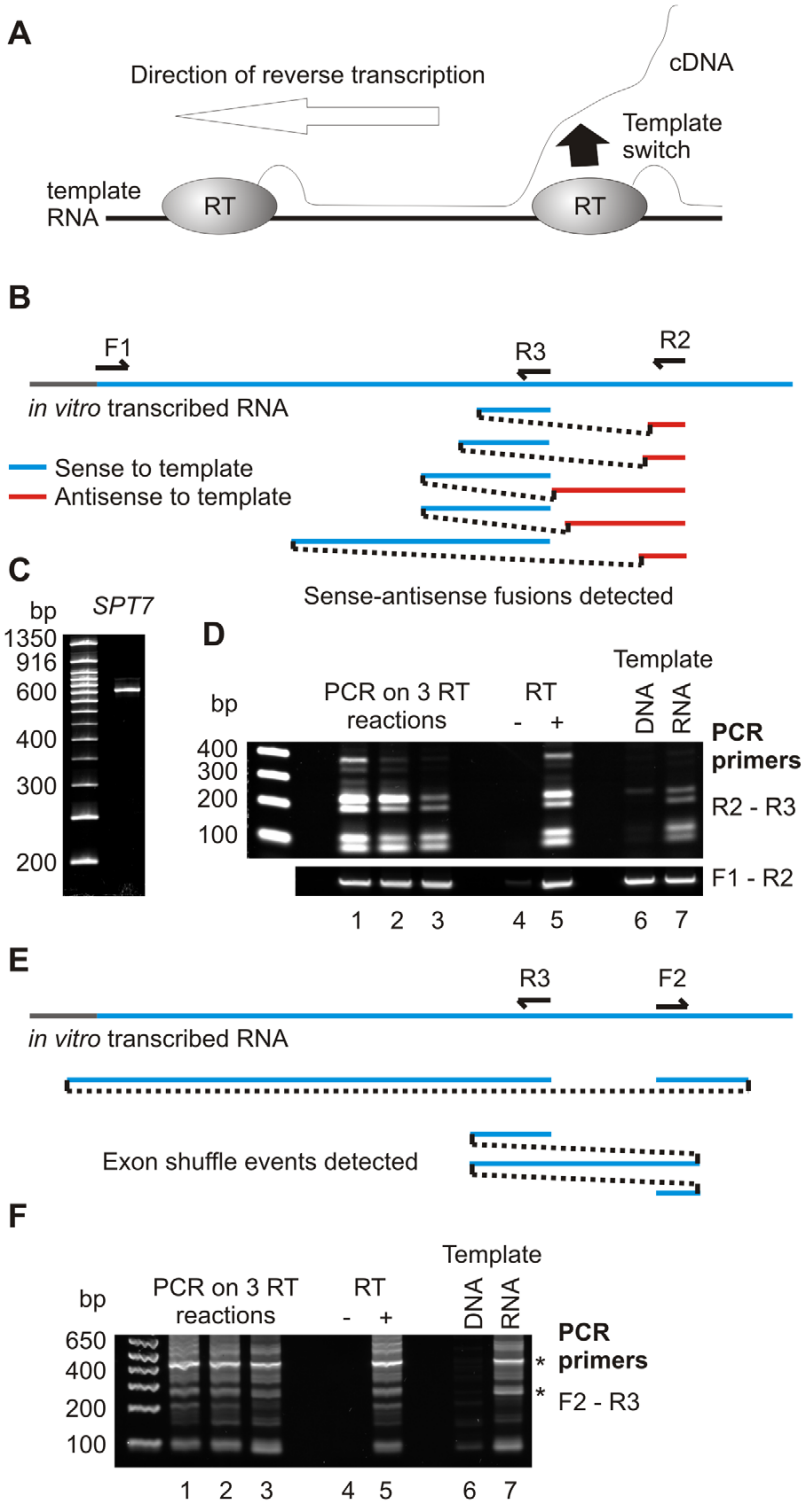
*In vitro* formation of sense-antisense fusions. A: Proposed mechanism of sense-antisense fusion formation. B: Schematic of *SPT7* RNA showing primer binding sites and observed sense-antisense fusions. C: Purified *SPT7* substrate. D: RT-PCR experiments performed on *SPT7* substrate performed as in Fig. 2C. Upper panel shows a 32 cycle PCR reaction, other panels show 25 cycles. E: Schematic of *SPT7* RNA showing primer binding sites and observed exon shuffling events. F: RT-PCR experiments performed as in d. Sequenced bands are indicated by *.

Ostensible, non-canonical trans-splicing events show a significant bias towards splicing between transcripts from the same locus. This has been taken to support their authenticity, since these sequences would be in close proximity *in vivo* but not in the RT reaction [14]. These events are classified as either exon shuffles (where exon order in the transcript differs from that in the genomic DNA), or fusions between sense mRNA and antisense non-coding RNA. However, the ability of reverse transcriptase to jump forward on a template (yielding apparent non-canonical cis-splicing), suggested that backwards jumps could generate exon shuffles. Moreover, trans-splicing between sense and antisense transcripts could be formed by a template switch from the RNA to the cDNA being produced by another RT on the same RNA (Fig. 3A).

To test these possibilities, we arbitrarily selected another yeast clone, GenBank sequence T37598, representing a sense-antisense fusion produced from the *SPT7* locus (Fig. 3B). As before, the region surrounding the apparent splice site was amplified from genomic DNA, cloned, transcribed, purified (Fig. 3C), and diluted with HeLa RNA prior to reverse transcription. To detect sense-antisense fusions, PCR reactions were performed using two primers complementary to the same DNA strand. This consistently generated the same set of product bands (Fig. 3D lanes 1–3). Sequencing of prominent bands from two independent RT-PCR reactions revealed multiple sense-antisense fusion events (depicted in Fig. 3B). Formation of these products required RT enzyme and did not occur during PCR on a DNA template (Fig. 3D lanes 4–7). Therefore, sense-antisense fusion events readily and reproducibly occur during reverse transcription *in vitro*.

A different primer pair was designed to detect exon shuffling (Fig. 3E). This consistently detected multiple species from the same reverse transcription reactions (Fig. 3F). Sequencing of the two clearly defined bands (marked * in Fig. 3F) confirmed the occurrence of apparent exon shuffling events involving *SPT7* RNA (Fig. 3E). We conclude that both types of trans-splicing seen at a single locus can be readily reproduced on a purified template *in vitro* using reverse transcriptase.

**Figure 4:**
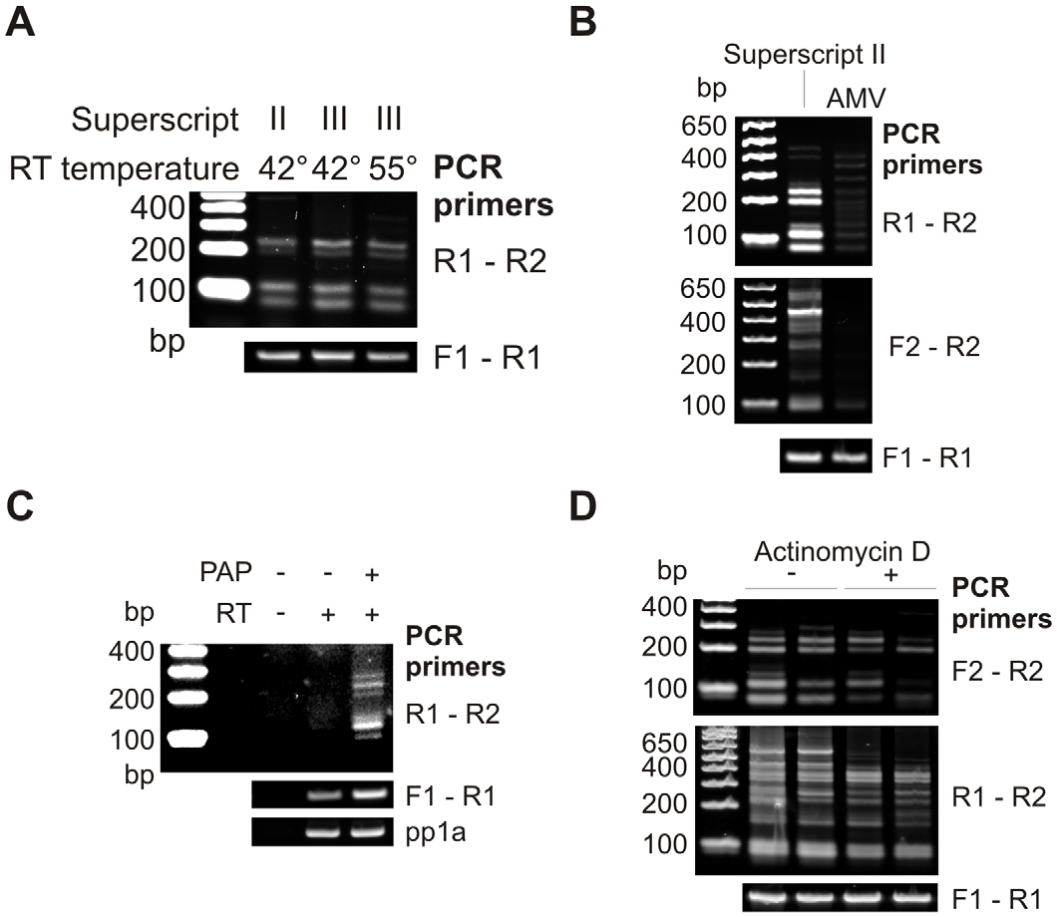
Generality of trans-splicing artifacts. A: PCR reactions for detecting sense-antisense fusion were performed on *SPT7* substrate RNA reverse transcribed with Superscript II or III at the given temperatures. Upper panel 30 cycle PCR reactions; lower panel 25 cycles. B: PCR reactions for detecting sense-antisense fusion (upper panel) and exon shuffling (middle panel) were performed on *SPT7* substrate RNA reverse transcribed with Superscript II or AMV. Upper panels 32 cycle PCR reactions; lower panel 25 cycles. C: RT-PCR using primers to detect sense-antisense fusions on poly(A) tailed RNA. Upper to lower panels show 32, 25 and 30 cycle PCRs. D: PCR reactions performed as in B using Superscript II, products from two RT reactions with and two RT reactions without 6 µg ml^−1^ Actinomycin D are shown.

As for the IGS1 intron, reverse transcription temperature did not alter the observance of sense-antisense fusions (Fig. 4A). In contrast, the prominent bands representing both types of trans-splicing at a single locus were not observed when AMV was substituted for Superscript II, although PCR products were still obtained, suggesting that some template switching occurs (Fig. 4B). However, the abundance of these products was too low for us to sequence, so we cannot rule out their arising from PCR mis-priming. The fact that prominent template-switching events were not obtained with AMV excludes the possibility that sense-antisense RNAs are produced by T7 RNA polymerase during transcription and survive the gel extraction step. Were this the case they should be amplified with similar efficiency by either RT.

Our proposed mechanism for the formation of sense-antisense fusions requires two RT enzymes to be active on the same RNA molecule. This will occur frequently if reverse transcription is primed from random hexamers, but is expected to be less common when oligo(dT) is used to prime synthesis from the poly(A) tail. To test the effect of this change, *SPT7* RNA was incubated with ATP in the presence or absence of E. coli poly(A) polymerase to add a poly(A) tail. These substrates were then used in vitro for reverse transcription as above but primed from oligo(dT). This produced the same pattern of products seen in previous experiments, which now depended on the presence of poly(A) polymerase (Fig. 4C). The pp1a control (a human mRNA present in the HeLa RNA) is presented to prove that RT efficiency was similar in the presence and absence of poly(A) polymerase. Note that some reverse transcription of the *SPT7* RNA still occurs in the absence of poly(A) polymerase due to priming of the oligo(dT) on short, encoded oligo(A) stretches in the substrate. These data show that template switching events detected in random hexamer primed RT reactions also occur during oligo(dT) primed cDNA synthesis.

Actinomycin D was at one time routinely added to RT reactions to suppress the formation of sense-antisense fusions caused by RT changing strand on hairpin structures at the 3′ end of the cDNA. This was, however, thought unnecessary after the introduction of RT lacking RNase H activity [19]. Although this ability is weak in RNase H deficient enzymes, it has recently been shown that actinomycin D can suppress the formation of some artifactual antisense RNAs [20]. Addition of actinomycin D to *SPT7* reactions reduced the aberrant products in some experiments, but clearly did not eliminate template switching (Fig. 4D).

Whereas the patterns of bands observed on gels following *in vitro* reactions were highly reproducible, sequencing of multiple products rarely revealed identical splice sites. Rather, the fusion sites varied by small numbers of nucleotides (Table S1). Similarly, the precise splice sites observed in the individual GenBank clones selected were not observed, but fusions were observed in close vicinity.

## Discussion

Reverse transcriptases have been invaluable tools in RNA analyses. It is, however, clear that these enzymes are error prone and the frequent introduction of point mutations by RT has been widely recognized. In contrast, their ability to generate artifacts that resemble splicing products remains largely unappreciated, despite being first reported many years ago [21]. One effect of template switching is the formation of sense-antisense fusion transcripts. This would lead to the detection of apparent antisense ncRNAs in high throughput experiments. Reported antisense ncRNAs that share the splicing pattern of the cognate sense mRNA are particularly likely to be artifacts [22]. Most template switching events are rare but the huge volume of transcriptome data currently being produced ensures that their contamination of cDNA databases will increase. Moreover, on particularly good substrates, such as the yeast IGS1 R ncRNA or the *FOXL2* mRNA [4], template switching occurs in a large fraction of cDNAs produced.

In our hands, the different template switching propensities of Superscript and AMV provided a useful diagnostic tool for identifying artifactual splicing events. Generally, however, our data show that all putative non-canonical splicing events and antisense ncRNAs require verification by non-RT based methods, e.g. northern blot or RNase protection, prior to their inclusion in further analyses. Other known methods to suppress template switching, notably elevated reverse transcription temperature and actinomycin treatment, failed to suppress *SPT7* sense-antisense fusion.

It is worth noting that some cases of trans-splicing observed in mammalian cells have been verified by non-reverse transcriptase based methods [23], [24]. However, these events occurred at canonical splice sites in contrast to the vast majority of reported trans-splicing events. We did not observe splicing at canonical splice sites in our *in vitro* system, and most events occurred between short direct repeats. However, direct repeats were not an absolute requirement, particularly for AMV, as we detected a number of trans-splicing events with little or no visible homology between donor and acceptor sequences.

Here we have confirmed a previous, controversial, report that reverse transcriptase can generate apparent trans-splicing [9]. We extended this analysis to prove that two other frequently encountered non-colinear splicing events, exon shuffling and sense-antisense fusion, can also be generated as reverse transcriptase artifacts. Furthermore we present a simple test for identifying many template switching events based on comparison of MMLV and AMV reverse transcription products.

## Materials and Methods

Substrates for *in vitro* assays were amplified from genomic DNA with Phusion (NEB) and cloned into pGEM-T (Promega). Oligonucleotides used were HXK1 F1/R1 for *HXK1*, KRE29 F1/R1 for *KRE29* and SPT7 F1/R1 for *SPT7*; sequences of oligonucleotides are given in Table 1. Plasmids were linearized with *Xho*I and 1 µg transcribed using T7 RNA polymerase (NEB) for 2 h at 37°C. Gels were run in 1x TBE, acrylamide gels contained 8 M urea. Gels were stained with SYBR Safe and imaged using a Fuji FLA5100 scanner. RNA was eluted from acrylamide gel slices by crushing and soaking for 4 h in 0.5 M NaOAc/1 mM EDTA/0.1% SDS, followed by phenol/chloroform extraction and ethanol precipitation with 1 µg glycogen. PCR reactions on reverse transcribed material were performed with Phire (NEB), details of cycle number are given in individual figure legends. Annealing temperature was 50°C for IGS1 and *HXK1*/*KRE29* PCR and 53°C for *SPT7* PCR. For poly(A) tailing, 50 ng RNA was incubated with 5U poly(A) polymerase (NEB) and 1 mM ATP, then cleaned on QIAQuick columns (QIAgen).

**Table 1:** Oligonucleotides used in this study.

IGS1 F	TGCAAAGATGGGTTGAAAGA
IGS1 R	TGTCCCCACTGTTCACTGTTCACTG
ASC1 F	TTCCATGATTATCTCTGGTTCCCG
ASC1 R	CATCTTGGGCAGACAAAGTG
HXKl F1	CACCTGGTCTTACCTCGAACT
HXK1 R1	CTCGAGATGCATAGTTCTTAGGTCTTCCAT
HXK1 R2	GGTATTTCGCTATCTTCCAACA
KRE29 F1	CGATGACGATGACGATGAGA
KRE29 R1	CTCGAGGGAACTTTTCCTTGCGATCT
SPT7 F1	CGACTCCCAATCTTATTTACTTG
SPT7 R1	CTCGAGCAGAAATCTGCTTTGTTTGTTG
SPT7 F2	TGACATAGTATACGAGGGAGTAAATAC
SPT7 R2	ATTTACTCCCTCGTATACTATGTCA
SPT7 R3	CTTCATTTCCATTTAAAGAGTGATC
pp1a F	CACCGTGTTCTTCGACATTG
pp1a R	TCGAGTTGTCCACAGTCAGC

Superscript II RT: 0.5 ng substrate RNA, 500 ng HeLa RNA (Invitrogen), 125 ng random hexamers and 0.5 µl 10 mM dNTPs in 6.5 µl total volume were denatured at 65°C for 5 min before 2 min on ice. 2 µl 5x first strand buffer and 1 µl of 0.1 M DTT were added followed by 0.5 µl (100 U) Superscript II (Invitrogen). Reactions were incubated 10 min at room temperature, 42°C for 50 min and 70°C for 15 min. For oligo(dT) priming, 250 ng oligo(dT)_18_ was added in place of hexamers, and reactions were heated to 42°C prior to enzyme addition. Superscript III reactions were performed as per manufacturer's instructions at the indicated temperatures. DNA template controls were cDNA from 500 ng HeLa RNA produced as above, with 0.5 ng gel purified *Xho*I-*Pvu*I fragments of the template plasmids. Where indicated, Actinomycin D (Calbiochem) was added to Superscript II RT reactions after the 65°C step at 6 µg ml^−1^ from a 1 mg ml^−1^ stock solution.

AMV RT: 0.5 ng RNA, 500 ng HeLa RNA (Invitrogen) and 125 ng random hexamers in total volume 8.25 µl were heated 5 min at 70° and 5 min on ice. 1.25 µl 10 mM dNTPs and 2.5 µl 5x buffer were added followed by 0.5 µl (5 U) AMV (Promega). Reactions were incubated for 1 h at 37°C.

## Supporting Information

Table S1 Sequencing information. All sequences obtained in this project are shown. Regions of the sequence have been colour coded red, green and blue to indicate that they emanate from different molecules or different regions of the same molecule. Overlapping regions are shown in purple. Sequences were obtained either by direct sequencing of band-purified PCR products, or for complex PCR products by sequencing multiple clones ligated in pGEM-T.
(0.04 MB DOC)
