# Supplementary material for: Apparent Non-Canonical Trans-Splicing Is Generated by Reverse Transcriptase In Vitro
Source: PLoS One. 2010 Aug 18;5(8):e12271. doi: 10.1371/journal.pone.0012271 (PMC2923612; doi:10.1371/journal.pone.0012271)
Supplement: Table S1 — Sequencing information. All sequences obtained in this project are shown. Regions of the sequence have been colour coded red, green and blue to indicate that they emanate from different molecules or different regions of the same molecule. Overlapping regions are shown in purple. Sequences were obtained either by direct sequencing of band-purified PCR products, or for complex PCR products by sequencing multiple clones ligated in pGEM-T. (0.04 MB DOC) [file pone.0012271.s001.doc]

**Table S1: Trans-spliced sequences**

All sequences obtained in this project are shown. Regions of the sequence have been colour coded red, green and blue to indicate that they emanate from different molecules or different regions of the same molecule. Overlapping regions are shown in purple. Sequences were obtained either by direct sequencing of band-purified PCR products, or for complex PCR products by sequencing multiple clones ligated in pGEM-T

*1) HXK1-KRE29­* fusions (Superscript II):

GAATAGCGACGAAGAGTTTTCTAGCCTAGAGAACAGCGATTTGAATCTTTCTGGTGCCAAGGCGGAGTCTGGAGACGATTTTGATCCAATCTTAAAAAGAACCATCATTTCCAAAAGAAAGGCACCAAGTAATAACGAGGATGAGGAGATTGTCAAGACACCCAGAAAATTAGTCAATTACGTACCTTTAAAAATTTTCAATCTAGGTGATAGCTTCGACGATACGATCACTACTACAGTGGCCAAGCTACAAGACTTGAAAAAAGAAATATTGGATTCACCTGGTCTTACCTCGAACTTTTCTTTTTACATCTTTTTCATTTTTTTACTTTTACCCCGCATCCNGNAAACCCCGGAAATTTTATTAGGAATAATTTATTTCCCGGNTGGAAATAAACCGGAAAAATGAANATTTANACNCTTTTAAGGNGTACGGGNTCCTGGCGTTTATCCCCTAAGTATACNGGTTGCCCCNGGCCNGATCNCNGTATAGCAGNGACNCGGGGGTTTCNGGAANAATGGCAGNCCCCTTTTGTTTTTCCGCATTGGGGGAGCTTCTTATGCCCCTGAACCCCNCTATTCTGCCCCTTTGAAACNCCCGCACGNGGGCCCCGTTTGTTGGAAGATAGCGAAATACCTTACTGGAGCAACCNGGAAAAATACTCTGGTTGCNAAAACCNACNAAAGAAAAAATGGAANACCTAANAACT

TTTTGCACCAGAGGTATTTTTCCTGGGTTGCTCCAGTAAGGTATTTCGCTTATCTTCAACCATAAGGGGCACACGNTGCGGGAGTTTCAAAGGGGCAGAATAGCGGGGTTCAGGGGCATAAGAAGCTCACACAATGCGGAAAAACAAAAGGGGACTGCCATTCTTCCTGAAACCCACGCGTCACTGCTATACTGAGATCTGGCCAGGGGCAACCAGTATACTTAGTGGATAAACGACAGGAACACGTACTCCTTAAAAGCGTCTAAATCTTCATTTTTCCGGTTTATTTCCAACCGGGAAATAAATTATTCCTAATAAAATTTCCGGGGTTTGCAGGATGCGGGGTAAAAGTAAAAAANTGAAAAAGATGTAAAAAGAAAAGTTCGAGGTAAGACCAGGTGAATCCNATATTTCTTTTTTCAAGTCTTGNAGATTGGNCNNTGGNATNNTGGTCCNTTCCGCNAAANTTTNNCNTNNNNTGNAAATTTTTAAAGGNCCANTCAATGTGNANTATTTNTCNGGGTGGTTTTGNAAATTNCCCCCNTNNCTTCNGTATNTTCTTGGGNGGCTTTTTNTTTGGAAAANNGTGGNTNCTCTTTTTANANATTGGATTNNAANTTCCCTNTNCANGACNTTCNCTCTNTNNGTNANCNTNTNANTTTTTCAANTGNNTTTGTTTTTNTTNGGNNTTTGAAAATTTNTTNTCTNNCCNNTTTTTNNTNNTTNTCCCTNTTTN

2) *HXK1-KRE29­* fusions (AMV):

AGGGGCAGAATAGTGGGGTTCAGGGGCATAAGAAGCTCACACAATGCGGAAAAACAAAAGGGGACTGCCATTCTTCCTGAAACCCACGCGNTCACTGCTATACTGAGATCTGGCCAGGGGCAACCAGTATACTTAGGATAACTAATAAAGTTGGGATGCACCTTCTCATCGTCATCGTCATCGAACA

GTTTGAATAGCGACGAAGAGTTTTCTAGCCTAGAGAACAGCGATTTGAATCTTTCTGGTGCCAAGGCGGAGTCTGGAGACGATTTTGATCCAATCTTAAAAAGAACCATCATTTCCAAAAGAAAGGCACCTGTGCCCCGGTTTGTTGGAAGATAGCGAAATACCA

GATCCGGAGACGACAGNTTTGAATAGCGACGAAGAGTTTTCTAGCCTAGAGAACTTTTCTTTTTACATCTTTTTCATTTTTTTACTTTTACCCCGCATCCTGCAAACCCCGGAAATTTTATTAGGAATAATTTATTTCCCGGTTGGAAATAAACCGGAAAAATGAAGATTTAGACGCTTTTAAGGAGTACGTGTTCCTGTCGTTTATCCACTAAGTATACTGGTTGCCCCTGGCCAGATCTCAGTATAGCAGTGACGCGTGGGTTTCAGGAAGAATGGCAGTCCCCTTTTGTTTTTCCGCATTGTGTGAGCTTCTTATGCCCCTGAACCCCACTATTCTGCCCCTTTGAAACTCCCGCACGTGTACCCCGTTTGTTGGAAGATAGCGGAAAATACCA

TCGATGACGATGACGATGAGAAGGTGCATCCCAACTTTATTAGTTTATCCACTAAGTATACTGGTTGCCCCTGGCCAGATCTCAGTATAGCAGTGACGCGTGGGTTTCAGGAAGAATGGCAGTCCCCTTTTGTTTTTCCGCATTGCGTGAGCTTCTTATGCCCCTGAACCCCACTATTCTGCCCCTTTGAAACTCCCGCACGTGTGCCCCGTTTGTTGGAAGATAGCGAAATACCA

TCGATGACGATGACGATGAGAAGGTTTATCCACTAAGTATACTGGTTGCCCCTGGCCAGATCTCAGTATAGCAGTGACGCGTGGGTTTCAGGAAGAATGGCAGTCCCCTTTTGTTTTTCCGCATTGTGTGAGCTTCTTATGCCCCTGAACCCCACTATTCTGCCCCTTTGAAACTCCCGCACGTGTGCCCCGTTTGTTGGAAGATAGCGAAA

TCGATGACGATGACGATGAGAAGGTGCATCCCAACTTTATTAGTGATCCTGAGAACGACAGTTTGAATAGCGACGAAGAGTTTTTTAGCCTAGAGAACTTTTCTTTTTACATCTTTTTCATTTTTTTACTTTTACCCCGCATCCTGCAAACCCCGGAAATTTTATTAGGAATAATTTATTTCCCGGTTGGAAATAAACCGGAAAAATGAAGATTTAGACGCTTTTAAGGAGTACGTGTTCCTGTCGTTTATCCACTAAGTATACTGGTTGCCCCTGGCCAGATCTCAGTATAGCAGTGACGCGTGGGCTTCAGGAAGAATGGCAGTCCCCTTTTGTTTTTCCGCATTGTGTGAGCTTCTTATGCCCCTGAACCCCACTATTCTGCCCCTTTGAAACTCCCGCACGTGTGCCCCGTTTGTTGGAAGATAGCGAAATACCA

TCGATGACGATGACGATGAGAAGGTGCATCCCAACTTTATTAGTGATCCTGAGAACGACAGTTTGAATAGCGACGAAGAGTTTTCTAGCCTAGAGAACTTTTCTTTTTACATCTTTTTCATTTTTTTACTTTTACCCCGCATCCTGCAAACCCCGGAAATTTTATTAGGAATAATTTATTTCCCGGTTGGAAATAAACCGGAAAAATGAAGATTTAGACGCTTTTAAGGAGTACGTGTTCCTGTCGTTTATCCACTAAGTATACTGGTTGCCCCTGGCCAGATCTCAGTATAGTAGTGACGTGTGGGTTTCAGGAAGACTGGCAGTCCCCTTTTGTTTTTCCGCATTGTGTGAGCTTCTTATGCCCCTGAACCCCACTATTCTGCCCCTTTGAAACTCCCGCACGTGTGCCCCGTTTGTTGGAAGATAGCGAAATACCA

TCGATGACGATGACGATGAGAAGGTGCATCCCAACTTTATTAGTGATCCTGAGAACGACAGTTTGAATAGCGACGAAGAGTTTTCTAGCCTAGAGAACAGCGATTTGAATCCCCCTGGCCAGATCTCATTATAGCAGTGACGCGTGGGTTTCAGGAAGAATTGCAGTCCCCTTTTGTTTTTCCGCATTGTGTGAGCTTCTTATGCCCCTGAACCCCACTATTCTGCCCCTTCGAAACTCCCGCACGTGTGCCCCGTTTGTTGGAAGATAGCGAAATACCA

3) *SPT7* sense-antisense fusions:

TATTCCTGGTGGGAATCTGGGTATCATCTAACTCAATATCATTGGGGTTGCTTTTCAAAATTCCATTATGAAAAATGGCTTTGGAACAGGTACCTAAAACAGGAAGACGATGACCAATTGCAGTTTCATAATGATCACTCTTTAAATGGAAATGAAGAGA

ATTTACTCCCTCGTATACTATGNTCAGGAAGACGATGACCAATTGCAGTTTCATAATGATCACTCTTTAAATGGAAATGAAG

ATTTACTCCCTCGTATACTATGTCAGGAATGGCNCTTACTAATATCATATTCCTGTAGGAATCTGGTATCATCTAACTCAATATCATTGGGTTGCTTTTCAAAATTCCATTATGAAAAATGGCTTTGGAACAGTACTAAAACAGGAAGACGATGACCAATTGCAGTTTCATAATGATCACTCTTTAAATGGAAATGAAG

ATTTACTCCCTCGTATACTATGTCAGGAATGGCTTTGGAACAGTACTAAAACAGGAAGACGATGACCAATTGCAGTTTCATAATGATCACTCTTTAAATGGAAATGAAG

TATTTACTCCCTCGTATACTATGTCAGGAATGGCTTTGGAACAGTACTAAAACAGGAAGACGATGACCAATTGCAGTTTCATAATGATCACTCTTTAAATGGAAATGAAGA

TATTTACTCCCTCGTATACTATGTCAGGAATGGCTTTGGAACAGTACTAAAACAGGAAGACGATGACCAATTGCAGTTTCATAATGATCACTCTTTAAATGGAAATGAAGA

TATTTACTCCCTCGTATACTATGTCAGGAATGGCTTTGGAACAGTACTAAAACAGGAAGACGATGACCAATTGCAGTTTCATAATGATCACTCTTTAAATGGAAATGAAGA

TATTTACTCCCTCGTATACTATGTCAGGAATGGCGTTACTAATATCATATTCCTGTAGGAATCTGGTATCATCTAACTCAATATCATTGGGTTGCTTTTCACAAGCTTTAGAATCATATCGTCAAAAAATAGAGCAAAATTCCATTATGAAAAATGGCTTTGGAACAGTACTAAAACAGGAAGACAATGACCAATTGCAGTTTCATAATGATCACTCTTTAAATGGAAATGAAGA

TATTTACTCCCTCGTATACTATGTCAGGAAGACGATGACCAATTGCAGTTTCATAATGATCACTCTTTAAATGGAAATGAAGA

4) *SPT7* exon skipping:

CATTGCAGGTTTCATAATGATCACTCTTTAAATGGAAATGAAGCTTTTGAAAAGCAACCCAATGATATTGAGTTAGATGATACCAGATTCCTACAGGAATATGATATTAGTAACGCCATTCCTGACATAGTATACGAGGGAGTAAATACTAAAACAGGAAGACGATGACCAATTGCAGTTTCATAATGATCACTCTTTAAATGGAAATGAAG

TCTTCATTTCCATTTAAAGAGTGATCATTATGAAACTGCAATTGGTCATCGTCTTCCATCTTGTCTAATGTTTTAGTATTTACTCCCTCGTATACTATGTCAGGAATGGCGTTACTAATATCATATTCCTGTAGGAATCTGGTATCATCTAACTCAATATCATTGGGTTGCTTTTCAAAAGCTTCATTTCCATTTAAAGAGTGATCATTATGAAACTGCAATTGGTCATCGTCTTCCATCTTGTCTAATGTTTTAGTATTTACTCCCTCGTATACTATGTCAA

TCTTCATTTCCTTTAAAGAGTGATCATTATGAAACTGCAATTGGTCATCGTCTTCCTGTTTTAGTACTGTTCCAAAGCCATTTTTCATAATGGAATTTTGCTCTATTTTTTGACGATATGATTCTAAAGCTTTCTGCTCTTTATATTCAAGAAAAAGCTGGTCGAACCTTTTCATTCTTTGTGGGTTTTTCAAAAACGCCTCTGAATCACTATTTAATTTTCCATTTTTAAAATATTCAGTTCTTTTTAAGCAAATTTCCGCACGAACTTTGGCAGTTACAGTTTTGTTTGTTGATACCATTTTGAAGCATGCGGTCCACGGAAGCGTCTTCCATCTTGTCTAATGTTTTAGTATTTACTCCCTCGTATACTATGTCA

TNTTCANTTCCANNTAAAGAGTGATCATTATGAANNNGCAANNGGTNNTCGTCCNCCTGCATTAGTANTGGGCCAAAGCCATTNNNCATAANGGNATTATGCNNTATTCCTTGACGATANGATTCNNAAGCTTTNTGCNCNNTATANGCAAGAAAAAGNTNGGCGANCCTTTTCNTNCNGGGTGGGTTNNTCAAAAACGCCTCTGAATCANTATNTAAGTTNTCCANTTTNGNANTAGTCAGTTCNGGTNAGCAAATNNCCGCNCGAACNGTGGCAGTAACAGTTTTCCATCTTGTCTAATGTTTTAGTATTTACTCCCTCGTATACTATGTCA

TCTTCATTTCCATTTAAAGAGTGATCATTATGAAACTGCAATTGGTCATCGTCTTCCTGTTTTAGTACTGTTCCAAAGCCATTTTTCATAATGGAATTTTGCTCTATTTTTTGACGATATGATTCTAAAGCTTTCTGCTCTTTATATTCAAGAAAAAGCTGGTCGAACCTTTTCATTCTTTGTGGGTTTTTCAAAAACGCCTCTGAATCACTATTTAATTTTCCATTTTTAAAATATTCAGTTCTTTTTAAGCAAATTTCCGCACGAACTTTGGCAGTTACAGTTTTCCACACGGATATTTCCAAATCGTCTCTATCGTCATCCTTTTTGTCTAATGTTTTAGTATTTACTCCCTCGTATACTATGTCA

TCTTCATTTCCATTTAAAGAGTGATCATTATGAAACTGCAATTGGTCATCGTCTTCCTGTTTTAGTACTGTTCCAAAGCCATTTTTCATAATGGAATTTTGCTCTATTTTT GACGATATGATTCTAAAGCTTTCTGCTCTTTATATTCAAGAAAAAGCTGGTCGAACCTTTTCATTCTTTGTGGGTTTTTCAAAAACGCCTCTGAATCACTATTTAATTTTCCATTTTTAAAATATTCAGTTCTTTTTAAGCAAATTTCCGCACGAACTTTGGCAGTTACAGTTTTCCACACGGATATTTCCAAATCGTCTCTATCGTCATCCTTTTCAAGTAAATAAGATTGGGAGTCGAATCCCGCGGCCATGGCGGCCGGGAGCATGCGGTCCACGGAAGCGTCTTCCATCTTGTCTAATGTTTTAGTATTTACTCCCTCGTATACTATGTCA

NB: underlined sequence is from pGEM-T between the MSC and T7 promotor
